# Therapeutic target database update 2014: a resource for targeted therapeutics

**DOI:** 10.1093/nar/gkt1129

**Published:** 2013-11-20

**Authors:** Chu Qin, Cheng Zhang, Feng Zhu, Feng Xu, Shang Ying Chen, Peng Zhang, Ying Hong Li, Sheng Yong Yang, Yu Quan Wei, Lin Tao, Yu Zong Chen

**Affiliations:** ^1^Bioinformatics and Drug Design Group, Department of Pharmacy, and Center for Computational Science and Engineering, National University of Singapore, 117543 Singapore, ^2^Molecular Medicine Research Center, State Key Laboratory of Biotherapy, West China Hospital, West China School of Medicine, Sichuan University, Chengdu, China, ^3^NUS Graduate School for Integrative Sciences and Engineering, National University of Singapore, 117456, Singapore, ^4^College of Pharmacy and Tianjin Key Laboratory of Molecular Drug Research, Nankai University, Tianjin 300071, People’s Republic of China, ^5^State Key Laboratory of Medicinal Chemistry & Biology, Tianjin International Joint Academy of Biotechnology & Medicine, Tianjin 300457, People’s Republic of China, ^6^Computation and Systems Biology, Singapore-MIT Alliance, National University of Singapore, Singapore and ^7^Innovative Drug Research Centre and College of Chemistry and Chemical Engineering, Chongqing University, Chongqing, People’s Republic of China

## Abstract

Here we describe an update of the Therapeutic Target Database (http://bidd.nus.edu.sg/group/ttd/ttd.asp) for better serving the bench-to-clinic communities and for enabling more convenient data access, processing and exchange. Extensive efforts from the research, industry, clinical, regulatory and management communities have been collectively directed at the discovery, investigation, application, monitoring and management of targeted therapeutics. Increasing efforts have been directed at the development of stratified and personalized medicines. These efforts may be facilitated by the knowledge of the efficacy targets and biomarkers of targeted therapeutics. Therefore, we added search tools for using the International Classification of Disease ICD-10-CM and ICD-9-CM codes to retrieve the target, biomarker and drug information (currently enabling the search of almost 900 targets, 1800 biomarkers and 6000 drugs related to 900 disease conditions). We added information of almost 1800 biomarkers for 300 disease conditions and 200 drug scaffolds for 700 drugs. We significantly expanded Therapeutic Target Database data contents to cover >2300 targets (388 successful and 461 clinical trial targets), 20 600 drugs (2003 approved and 3147 clinical trial drugs), 20 000 multitarget agents against almost 400 target-pairs and the activity data of 1400 agents against 300 cell lines.

## INTRODUCTION

Modern drug development has been primarily focused on targeted therapeutics (1–3) with increasing movement toward stratified and personalized medicines (4–6). Extensive efforts from the research, industry, clinical, regulatory and management communities and the chemistry, biology, pharmaceutics and medicine disciplines have been collectively directed at the discovery, investigation, application, monitoring and management of targeted therapeutics and biomarkers (4,7–10). The knowledge of the efficacy targets and biomarkers is useful not only for the discovery and development of targeted therapeutics (11,12) but also for facilitating the development and practice of stratified and personalized medicines (4,13,14).

In particular, the information of targeted therapeutics and biomarkers may be potentially incorporated into the widely used disease classification systems for more refined classification of disease subclasses and patient subpopulations responsive to a particular treatment so as to better facilitate the diagnosis, prescription, monitoring and management of patient care in stratified and personalized medicines. Although the information about targeted therapeutics and biomarkers can be obtained from the established drug (15), efficacy target (16) and biomarker (17–19) databases, the data retrieval tools of these databases are not specifically designed for optimally supporting such tasks. There is a need to enable data retrieval by using the widely used International Classification of Diseases (ICD) codes (20,21) for facilitating broader, more convenient and automatic data access, processing and exchange by the bench-to-clinic communities, particularly non-domain experts.

To better serve the multiple bench-to-clinic communities and to facilitate the development and practice of stratified and personalized medicines, we made several major improvements to the Therapeutic Target Database (TTD, http://bidd.nus.edu.sg/group/ttd/ttd.asp). First, we added information and search tools based on the ICD codes (22,23) for searching the targets, biomarkers, drugs and other TTD data related to various disease conditions. For more extensive coverage of potential biomarkers and for enabling their convenient access by the ICD codes, we added a significantly higher number (1755) of literature-reported biomarkers for more variety of disease conditions (365) than those in the existing biomarker databases that primarily focus on molecular biomarkers of specific disease classes (17,19) or clinically prioritized sets (18). We also added information and enabled the search of TTD data via drug scaffold names (227 scaffolds for 736 drugs and leads) for facilitating the search of the drugs, targets and diseases related to specific molecular scaffolds. Moreover, we added the Anatomical Therapeutic Chemical (ATC) Classification System codes for 1521 approved drugs for supporting the convenient and automated access of clinical drug data (24).

By using the literature search methods described in our earlier article (16), we also significantly expanded TTD contents to include 388 successful, 461 clinical trial and 1467 research targets; 2003 approved (1008 nature product derived), 3147 clinical trial, 498 discontinued clinical trial and 14 856 experimental drugs, 20 818 multitarget agents against 385 target-pairs and the activity data of 1436 drugs against 274 cell lines. These are compared with the 364 successful, 286 clinical trial and 1331 research targets; 1540 approved (939 natural product derived), 1423 clinical trial, 345 discontinued clinical trial and 14 853 experimental drugs, and 3681 multitarget agents active against 108 target pairs in our last update (16). The statistics of our updated data is summarized in Table 1.

**Table 1.** Statistics of the drug targets, drugs and their structure and potency data in 2014 version of TTD database

Category	Item	2014 Update	2012 Update
Statistics of drug targets	Number of all targets	2360	2025
	Number of successful targets	388	364
	Number of clinical trial targets	461	286
	Number of research targets	1467	1331
Statistics of drugs	Number of all drugs	20 667	17 816
	Number of approved drugs (no of natural product derived drugs)	2003 (1008)	1540 (939)
	Number of clinical trial drugs (no of natural product derived drugs)	3147 (369)	1423 (369)
	Number of discontinued drugs	498	345
	Number of pre-clinical drugs	163	165
	Number of experimental drugs	14 856	14 853
	Number of multitarget agents	20 818	3681
	Number of drug combinations	115	115
Statistics of drugs with available structure or sequence data	Number of small molecular drugs with available structure	17 012	14 170
	Number of antisense drugs with available sequence data	652	652
Statistics of drugs with activity data or structure-activity relationship	Number of agents with potency data against target	11 810	11 810
	Number of agents with potency data against a disease model such as a cell-line, *ex vivo*, *in vivo* model	1753	497
	Number of quantitative structure-activity relationship qsar models (no of chemical types)	841 (228)	841 (228)

### International classification of diseases

ICD has been developed by the World Health Organization (WHO), sponsored by the United Nations, adopted by >110 countries and used by physicians, researchers, nurses, health workers, health information managers, policy makers, insurers and health program managers for defining and studying diseases, monitoring and managing health care and allocating resources (20,21). ICD codes have been regularly revised to the current version ICD-10 (20). But the previous version ICD-9 is still used by some organizations while proceeding with the transition to ICD-10 (the expected completion date for the transition to ICD-10 in the United States is October 1, 2014) (25). ICD-10 is composed of 68 000 alphanumeric codes as compared with the 13 000 numeric codes in ICD-9, thus offering more comprehensive coverage and better representation of medical conditions (20). A number of nations have developed their own adaptations of the ICD codes. For instance, the United States have developed ICD-9 and ICD-10 clinical modification ICD-9-CM (17 000 codes) and ICD-10-CM (155 000 codes) for covering additional morbidity details (26), which were used in TTD because of their more comprehensive coverage.

The ICD-9-CM and ICD-10-CM codes were matched to the TTD target, drug and biomarker entries by the following procedure. First, automated word match was conducted for matching the disease name or names of each TTD target, drug or biomarker entry with the disease descriptions of each ICD codes. Second, each of the fully or partially matched TTD entry was manually checked to either validate the match or to find the right ICD codes. Third, manual search was conducted for every non-matched TTD entries. So far, we were able to find the ICD codes for 785 targets and 3080 drugs related to 732 disease conditions. From the TTD ‘Search drugs and targets by disease or ICD identifier’ field, users can search TTD target and drug entries related to a specific disease or an ICD-9-CM or ICD-10-CM code. The TTD biomarker entries may also be searched by selecting an ICD-9-CM or ICD-10-CM code from the ‘Search for biomarkers’ field. Users may also download from the TTD download page the lists of TTD target, drug and biomarker entries with the corresponding ICD-9-CM and ICD-10-CM codes.

A new ICD version ICD-11 is in development and scheduled for endorsement by WHO in 2015 (WHO. The International Classification of Diseases 11th revision is due by 2015. Retrieved from http://www.who.int/classifications/icd/revision/en/), which offers more refined disease classifications based on more recent scientific understanding of the disease mechanisms. For instance, small cell lung cancer, which represents ∼13% of all lung cancer diagnoses (27), is not explicitly classified in the ICD-10 and earlier ICD versions but is now explicitly represented in the ICD-11 beta draft. Therefore, ICD-11 is expected to be more useful for developing a more refined disease classification system for stratified and personalized medicine. Effort will be made to upgrade TTD to the ICD-11 version on its official release.

### Biomarkers

Biomarkers have been developed as non-invasive tests for early detection and indication of disease risks, monitoring of disease progression and recurrence and classification of disease subtypes and patient subpopulations for providing the most appropriate treatments (28–30). As many therapies have been found to elicit markedly different clinical responses in individual patients (31,32), there is a particular need for more biomarkers capable of predicting drug response in individual patients, which has led to intensive efforts in the discovery of such biomarkers (4,33). Table 2 gives examples of the approved and clinically tested biomarkers for facilitating the prescription of a particular drug to specific patient subpopulation. Moreover, there are considerable interests in adopting the multimarker strategy for parallel evaluation of multiple existing and novel biomarkers in the diagnosis and prognostics of diseases and treatment responses in individual patients (34,35). These efforts may be facilitated by significantly expanding biomarker coverage in the biomarker databases. We, therefore, searched literature-reported biomarkers, mapped them to the ICD-9-CM and ICD-10-CM codes and added the relevant information and ICD code search tools in TTD.

**Table 2.** Examples of the approved and clinically tested biomarkers for facilitating the prescription of a particular drug to specific patient subpopulation

Disease	Therapeutic target	Biomarker for the targeted therapeutics	Patient subpopulation likely responsive to targeted therapeutics	Drug therapy specific for patient subpopulation
Acute promyelocytic leukemia (APL)	PML–RAR	PML–RAR (gene translocation)	APL with PML–RARα t(15:17) translocation	Arsenic Trioxide
Alzheimer’s	PPAR	apolipoprotein E and TOMM40 genotypes and age	Mild cognitive impairment due to Alzheimer’s disease	Pioglitazone
Breast cancer	HER2	HER2 (gene amplification)	HER2 amplified and/or over-expressed breast cancer	Trastuzumab
	Estrogen receptor	Estrogen receptor (protein expression)	ER overexpressed breast cancer	Tamoxifen
	PARP	BRCA1/2 (mutation)	Breast cancer defective in BRCA1 or BRCA2	Olaparib, veliparib
Cystic fibrosis	CFTR	CFTR G551D mutation	Cystic fibrosis patients with CFTR G551D mutation	Ivacaftor
Hepatitis C infection	HCV non-structural protein 3	IL28B rs12979860 genotype	HCV infected patients with IL28B rs12979860 genotype	Boceprevir
Melanoma	BRAF	BRAF V600E (mutation)	Melanoma with RAF V600E mutation	Vemurafenib, Dabrafenib
	MEK	BRAF mutations	Melanoma with RAF mutations	Trametinib
Post-menopausal osteoporosis	RANK ligand	Post-menopausal women with persistent total hip, femoral neck, or lumbar spine BMD T-scores −1.8 to −4.0, or clinical fracture	Post-menopausal osteoporosis at high risk for fractures	Denosumab

To broadly cover various types of biomarkers, we conducted comprehensive literature search in the PubMed database (36) by using combination of keywords ‘biomarker’, ‘clinical’, ‘patient’, ‘disease’, ‘drug’ and specific disease names. Additional sources such as the FDA website and the abstracts of the American society of clinical oncology were also systematically searched. Overall we collected 1755 biomarkers for 365 disease conditions, which include both process biomarkers (genetic mutations or alterations, gene amplification and levels of proteins, gene expression, microRNAs, small molecules, or metabolites that capture a molecular/biochemical aspect of disease pathogenesis and the biological responses to the disease process and/or treatment) and global biomarkers (such as tumor sizes, brain structures in neurodegeneration and shape of cells in anemia). These biomarkers may be searched in the ‘Search for biomarkers’ field by using keywords or by selecting an ICD-9-CM or ICD-10-CM code.

Based on the literature descriptions, our collected biomarkers were classified into one or more of the following 12 classes: associative (disease correlation), antecedent (pre-illness risk identification), detective (disease early stage detection), classification (disease categorization and patient assignment for differential treatment), differentiative (differentiation of related diseases), diagnostic (recognition of overt diseases), monitoring (monitoring of disease state or treatment response), pharmacodynamic (examination of the biological basis of drug response variations), pharmacogenomic (genomics-based prediction of drug response, adverse drug reaction and appropriate drug dose), prognostic (prediction of future disease course and response to therapy), surrogate (substitute of a clinical end point for predicting therapeutic benefit) and theragnostic (identification and monitoring of biochemical effects or mode of action of drug and downstream processes) classes.

Apart from the literature-reported biomarkers, the profiles of various known drug resistance mutations (37–39) and drug response regulators (e.g. the genes promoting drug bypass signaling (40,41) or hindering drug actions (42) have been studied for predicting drug resistance, which may be potentially explored as drug response biomarkers (43). Potential biomarkers, particularly multimarkers, have also been predicted from the genetic and gene expression data of patients by using such computational methods as the principal components analysis feature selection method (44), weighted voting classification feature selection method (45), hierarchical clustering feature selection method (46), differentially expressed genes method (47,48) and machine learning feature selection methods (49,50). These potential biomarkers may also be included in TTD and other biomarker databases for facilitating their future exploration.

### More refined classification of patient subpopulations for targeted therapeutics

From the examples of the approved and clinically tested drug response biomarkers in Table 2, it seems feasible to incorporate target and biomarker codes into the ICD codes for more refined classification of patient subpopulations responsive to a particular targeted therapy. However, many of the existing biomarkers are based on the profile of a single gene. For highly heterogenetic diseases such as cancers, single-gene biomarkers are highly limited in their coverage of drug escape mechanisms, and multimarkers may be needed for more sufficient coverage of drug escape mechanisms and for more accurate classification of patient subpopulations in stratified and personal medicines. For instance, BRAF^V600E^ inhibitor dabrafenib has shown improved therapeutic effect in BRAF^V600E^ metastatic melanoma patients (51) due in part to its specificity to BRAF^V600E^ tumors with a greater therapeutic window (52). However, drug resistance still emerges (51) partly due to tumor activation of several BRAF inhibitor escape pathways (52–54). Therefore, the use of a single-gene biomarker, BRAF^V600E^ mutation, is insufficient for predicting long-term drug response to dabrafenib therapy, and multimarkers are needed for adequately covering these and other active drug escape mechanisms.

### Drug scaffolds

The approved and clinical trial drugs are composed of a limited number of molecular scaffolds (55–57) in contrast to the high number of bioactive molecular scaffolds (58,59). For instance, many drugs have been derived from individual scaffold groups such as macrocycles (60), and 12 FDA-approved anticancer kinase inhibitor drugs (61,62) are grouped into three scaffold groups (63). Investigation and exploration of these highly privileged drug scaffolds are important for discovering new drug-like scaffolds, molecular analogs and drugs. To support the relevant efforts, we searched the literatures for the molecular scaffolds of the approved and clinical trial drugs or their drug leads. By using the combination of keywords drug name or alternative name, ‘scaffold’, ‘discovery’, ‘synthesis’ to search the Pubchem database (36), we found 210 scaffolds for 714 drugs and drug leads. Users can search the TTD drug and target entries related to a drug scaffold by keyword search or by selecting from the list of drug scaffold names in the ‘Search for drug scaffolds’ field.

### Remarks

The efforts in the discovery and application of targeted therapeutics increasingly involve collective efforts from multiple bench-to-clinic communities (1–3) and these efforts are increasingly directed at the development of stratified and personalized medicines (4–6). The drug, target, biomarker and other relevant chemical, biological, pharmaceutical and clinical data need to be more integrated and be made easily accessible by the multiple bench-to-clinic communities. These efforts may be partly facilitated by introducing into the relevant databases the ICD code-based data retrieval tools coupled to the other domain knowledge codes such as the codes of drugs (e.g. ATC codes), targets and biomarkers. Continuous efforts will be made to expand the linkage of the ICD and ATC codes to more complete sets of drugs, efficacy targets and biomarkers and to provide the latest and comprehensive information about the drugs, efficacy targets and biomarkers for better serving the multiple bench-to-clinic communities in their collective efforts for the discovery, investigation, application, monitoring and management of targeted therapeutics.

## FUNDING

Singapore Academic Research Fund [R-148-000-181-112]; National Natural Science Foundation of China [81202459]; Chongqing Natural Science Foundation [cstc2012jjA10116]; Fundamental Research Funds for the Central Universities [CQDXWL-2012-Z003]; Start up founding of Youth 100 Talents program of Chongqing University [0903005203176]; The Major State Basic Research Development Program of China [2013CB967204]. Funding for open access charge: National Natural Science Foundation of China [81202459].

*Conflict of interest statement*. None declared.
